# Serum CEACAM1 Elevation Correlates with Melanoma Progression and Failure to Respond to Adoptive Cell Transfer Immunotherapy

**DOI:** 10.1155/2015/902137

**Published:** 2015-11-25

**Authors:** R. Ortenberg, S. Sapoznik, D. Zippel, R. Shapira-Frommer, O. Itzhaki, A. Kubi, D. Zikich, M. J. Besser, J. Schachter, G. Markel

**Affiliations:** ^1^The Ella Lemelbaum Institute for Melanoma, Sheba Medical Center, 52621 Tel Hashomer, Israel; ^2^Talpiot Medical Leadership Program, Sheba Medical Center, 52621 Tel Hashomer, Israel; ^3^Clinical Microbiology and Immunology, Sackler School of Medicine, Tel Aviv University, 69102 Tel Aviv, Israel

## Abstract

Malignant melanoma is a devastating disease whose incidences are continuously rising. The recently approved antimelanoma therapies carry new hope for metastatic patients for the first time in decades. However, the clinical management of melanoma is severely hampered by the absence of effective screening tools. The expression of the CEACAM1 adhesion molecule on melanoma cells is a strong predictor of poor prognosis. Interestingly, a melanoma-secreted form of CEACAM1 (sCEACAM1) has recently emerged as a potential tumor biomarker. Here we add novel evidences supporting the prognostic role of serum CEACAM1 by using a mice xenograft model of human melanoma and showing a correlation between serum CEACAM1 and tumor burden. Moreover, we demonstrate that serum CEACAM1 is elevated over time in progressive melanoma patients who fail to respond to immunotherapy as opposed to responders and stable disease patients, thus proving a correlation between sCEACAM1, response to treatment, and clinical deterioration.

## 1. Introduction

The incidence of melanoma has more than doubled over the last two decades, making it one of the fastest rising cancers worldwide. When diagnosed at early stages, the disease is curable by surgical removal. Currently, however, the mortality rate is significantly higher than the 1.3% diagnosed with incurable metastatic disease at presentation, implying its metastatic potential (reviewed in [1, 2]). The clinical landscape of antimelanoma drugs has evolved remarkably over the last years by the generation of molecular targeted therapies (BRAF and MEK inhibitors) and immunotherapies (anti-CTLA4 and anti-PD1 antibodies) [3, 4].

The monitoring of melanoma patients relies mainly on physical examination, history taking, periodical imaging, and routine blood tests. There are no simple tests for monitoring melanoma patients in the outpatient setting and the available serum biomarkers (the most reliable and wildly used one being LDH) provide limited information [5, 6]. The rapid rise in melanoma prevalence, emerging era of antimelanoma therapies which are beneficial only for a subset of the patients, and the extraordinary ability of malignant melanoma to remain dormant before relapsing all emphasize the need for novel prognostic biomarkers for melanoma.

CEACAM1, an adhesion molecule belonging to the CEA (carcinoembryonic Ag) family, is a transmembrane glycoprotein expressed on epithelial, endothelial, and hematopoietic cells, where it regulates immune responses, insulin clearance, and neovascularization [7–9]. In healthy volunteers CEACAM1 expression can thus be detected mainly in the luminal side of epithelial cells forming ducts or glands in the visceral organs such as the small intestine, liver bile canaliculi, the kidney, and salivary gland and in hematopoietic cells such as neutrophils [10]. While downregulated in some cancers [11–14], CEACAM1 is elevated stepwise during the course of melanoma progression [15]. Its expression in melanoma strongly correlates with the development of metastases and poor survival, and its prognostic value is similar or even superior to that of the widely accepted Breslow score (determining tumor thickness at presentation) [16]. While it is expected that the pattern of nonhematological tissue-specific expression of CEACAM1 in melanoma patients would be similar to healthy donors, it has never been directly studied. We have previously shown that an unusual elevated level of CEACAM1-positive T cells and NK cells is found in the circulation of melanoma patients [17] and that CEACAM1 serves as immune evasion mechanism from NK and T cells [18–20]. Based on these findings, we have raised an anti-CEACAM1 blocking antibody that renders melanoma cells more vulnerable to cytotoxic immune cells both* in vitro* and* in vivo* and is a promising strategy for treating melanoma patients [4, 10].

While the therapeutic significance of anti-CEACAM1 therapy awaits further examination, a soluble form of CEACAM1 (sCEACAM1) was described in body fluids, including serum, bile, saliva, and seminal fluid [21–24]. The exact function of sCEACAM1 is still unknown. We reported that the secretion of sCEACAM1 from melanoma cells is an active process, which depends on protein synthesis and intact intracellular vesicular transport, and does not result merely from dead cells or shedding and is not correlated with surface membrane expression intensity [17].

Abnormal decreased levels of sCEACAM1 are found in TAP-2 deficient patients [21]. Elevated sCEACAM1 levels characterize several malignancies, among them are biliary diseases (i.e., obstructive jaundice, primary biliary cirrhosis, autoimmune hepatitis, and cholangiocarcinoma) [22, 24, 25], meningococcal sepsis [26], and, importantly, malignant diseases such as malignant melanoma [17, 27], pancreatic cancer [28, 29], bladder cancer [30], and non-small-cell lung cancer [31]. In melanoma, serum CEACAM1 is elevated in patients with evidence of disease as compared with patients with no evidence of disease or healthy controls, and its expression correlates with LDH, disease state, and decreased survival [17]. Moreover, following vaccination with modified autologous melanoma cells as postsurgical adjuvant therapy, the changes in postvaccination serum CEACAM1 correlate with overall survival and with the S100B melanoma marker [27]. Thus, serum CEACAM1 is a potential novel prognostic biomarker for melanoma progression and predication of response to treatment.

Here we study in xenograft models the correlations between human sCEACAM1 in mice sera and tumor burden, in various scenarios of disease progression, surgical removal of tumor mass, and relapse. The potential of serum CEACAM1 to reflect or predict response to therapy of metastatic melanoma patients with adoptive cell transfer of tumor infiltrating lymphocytes was also tested.

## 2. Materials and Methods

### 2.1. Healthy Volunteers and Melanoma Patients

We used a cohort of 47 healthy volunteers. 27 patients were males (57%). 14 donors were <40 years of age, nine were 41–50, thirteen were 51–61, and eleven were 61+ years of age. We used a cohort of 27 AJCC stage IV malignant melanoma patients, showing no other signs of malignancy or health disorders. All patients were treated with tumor infiltrating lymphocytes (TIL) immunotherapy [32, 33] after being refractory to other treatments. All TIL treatments were based on the “young TIL” protocol, except for one patient who was treated with the “selected TIL” protocol [32, 33]. One of the patients was given two sequential TIL treatments with 13 months apart and partially responded to each of them. These treatments were considered as two different sets in our analysis, which consisted of 28 sets of pre- and posttherapy values. Twenty patients were males (74%). Five patients were <40 years of age, seven were 41–50, twelve were 51–60, and three were 61+ years of age.

### 2.2. Clinical Study Design

A longitudinal retrospective clinical study was performed, in which patients' sera were collected and analyzed for sCEACAM1 levels by ELISA. Samples were obtained in the Sheba melanoma clinic at 3 time points: on decision to go for TIL therapy, which was 43–103 days before TIL treatment (median: 49.5 days), at first posttreatment evaluation which was 48–102 days after treatment (median: 86.5 days), and at second posttreatment evaluation which was 330–552 days after treatment (median: 348 days). Samples were collected from May 2006 through March 2011. The follow-up period was 70–1519 days (median: 194.5 days). The clinical data was analyzed at June 2015. Following evaluation for radiological response, the patients were described according to RECIST 1.0 criteria as progressive disease patients (PD, *n* = 10), stable disease (SD, *n* = 6), or responders that included the partial (PR, 7 patients; 8 data sets) or complete response (CR, *n* = 4) patients. Notably, the evaluations were not changed between the 1st and the 2nd posttreatment time points. All patients gave written informed consent prior to their participation in this study. This study was approved by the Israel Ministry of Health.

### 2.3. Clinical Samples Handling

Blood samples were obtained from healthy donors and melanoma patients by venopuncture and standard handling procedures. 10 mL of blood was collected in heparinized tubes (BD Biosciences) and then centrifuged at 590 g for 15 min in room temperature to obtain plasma. All plasma samples were collected and divided into aliquots and frozen in −80°C until analyzed. Anonymous samples (marked only with ID number) were linked only to clinical-pathological data.

### 2.4. Melanoma Xenograft Model

In the xenograft model we use the primary melanoma culture 009mel which was developed from surgically resected tumor and was established and grown as previously described [32]. 3 × 10^6^ 009 melanoma cells were injected subcutaneously to the thigh of 7-8-week-old SCID-NOD mice to create human melanoma xenografts. Mice were monitored once weekly for tumor volume by caliper measurements. Tumor volume was calculated as (small diameter)^2^  × (large diameter)/2. Mice were sacrificed when showing a reduction of more than 20% in body weight or when tumor volumes reached 3800 mm^3^. For tumor excision experiments, 20 mice were used. When tumors reached a volume of 500 mm^3^, mice were randomized into 2 equal experimental groups, with one of them undergoing tumor excision (which was complete in 3 mice and partial in 7 mice) and the other sham surgery. All animal work was performed following approval of Sheba Medical Center IRB (861/2013).

### 2.5. Mice Sera Samples

Blood samples were collected once weekly from the retroorbital plexus of anesthetized mice as described [34]. Anesthesia was induced by placing each mouse in an inhalation chamber with 4% isoflurane (Abbott). The volume of each blood sample was ~250 *μ*L and at no time did this volume exceed that recommended for mice in regard to body weight and recovery time. Mice were allowed to recover completely after each bleeding session and were observed daily for signs of pain and discomfort. Blood samples were deposited in heparinized tubes (BD Biosciences), centrifuged for serum separation (15 min at 590 g at room temperature), and frozen at −80°C until a later, technically convenient point. Serum hemolysis was evaluated by direct observation.

### 2.6. Anti-Human CEACAM1-Based ELISA

All sera used were thawed at once and subjected to anti-human CEACAM1 ELISA as described [17], using MRG1 as the capture antibody. The anti-human CEACAM1 antibody MRG1 was generated by us as described [10]. Each sample was tested in triplicate repeats.

### 2.7. LDH Evaluation in Sera Samples

LDH was evaluated in sera samples from 26 out of the 27 patients participating in the cohort, using kinetic UV quantitative evaluation on Beckman Coulters AU analyzers (Beckman Coulter LDH reagent OSR6128). LDH tests were performed on sera samples obtained at the same time points as used for serum CEACAM1 evaluations.

## 3. Results

### 3.1. Serum CEACAM1 Correlates with Melanoma Tumor Volume in Xenografted Mice

**Figure 1:**
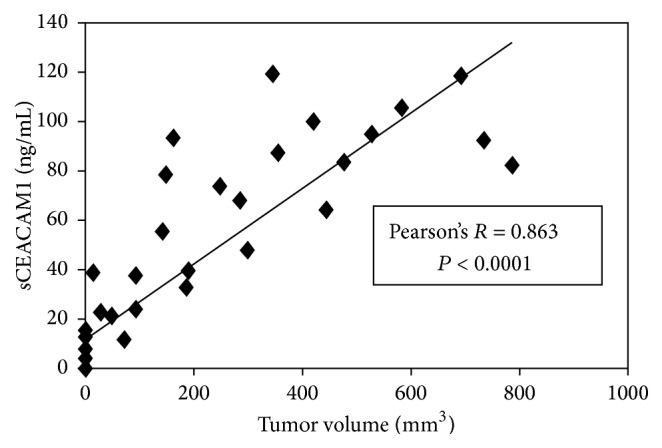
sCEACAM1 directly correlates with tumor burden in mice xenografts. 10 SCID-NOD mice were subcutaneously xenografted with human 009mel. Starting from the day of injection and through 3 weeks, serum samples were collected for further anti-human CEACAM1 ELISA and tumor volume was measured once weekly. The graph shows sCEACAM1 levels versus tumor volume.

We have previously shown that CEACAM1 in its soluble form (sCEACAM1) is secreted from several primary cultures and cell lines of human melanoma [17]. The concentration of secreted CEACAM1 was found to be proportional to the number of melanoma cells seeded in culture [17]. In order to test whether serum CEACAM1 correlates with melanoma mass* in vivo* we used a xenograft model in which primary human melanoma cells are injected subcutaneously to SCID-NOD mice [10] and serum CEACAM1 is measured by anti-human CEACAM1-based ELISA. 10 mice were thus injected to the thigh with 3 × 10^6^ 009mel cells. Once a week, starting from the day of injection and for 3 weeks, tumor volumes were measured by caliper and sera were collected, frozen, and stored until all samples were gathered and used for ELISA (Figure 1). Notably, murine serum CEACAM1 was not recognizable by the anti-human CEACAM1 antibody ([10] and tumor volume zero point in Figure 1). In contrast, human serum CEACAM1 strongly and directly correlated with tumor burden (Pearson's *R* = 0.863, *P* value < 0.0001) and was detectable even at minor tumor volume of 14 mm^3^, implying its high sensitivity (Figure 1).

### 3.2. Tumor Excision and Recurrence in Xenografts Are Readily Reflected by sCEACAM1

**Figure 2:**
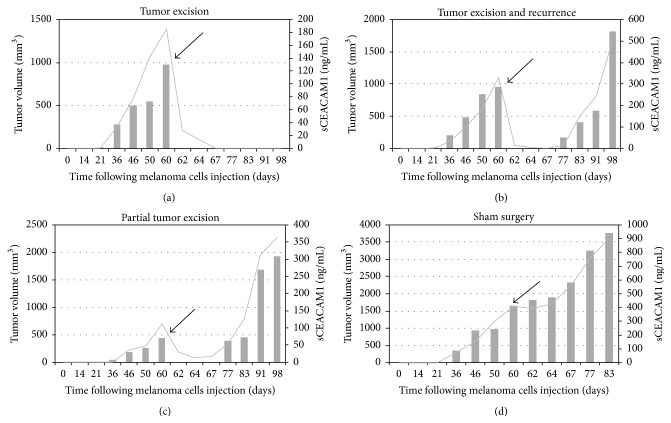
sCEACAM1 sensitively reflects tumor excision and recurrence in mice. 20 SCID-NOD mice were subjected to subcutaneous injection of 009mel cells. When tumors reached ~500 mm^3^, they were excised from half of the mice in a complete ((a), *n* = 3) or partial ((b)-(c), *n* = 7) manner while the other mice underwent sham surgery ((d), *n* = 10). In some of the mice recurrence of tumor occurred ((c), *n* = 4). sCEACAM1 levels (depicted by a solid line) as well as tumors volumes (gray bars) were periodically measured and plotted against time. The arrow denotes the excision time point. The experiment was repeated two independent times. Shown are results from one representative mouse from each group.

We next tested whether serum CEACAM1 levels follow the clinical scenarios of tumor excision and recurrence. 20 SCID-NOD mice were injected as described above with 009mel cells. When tumors reached ~500 mm^3^, tumor was excised from half of the mice in a complete or nearly-complete manner, while the other half served as a control group and underwent sham surgery. Tumors were measured and sera collected periodically as in Figure 1, except for the week of surgery, in which sera were collected three times from each mouse. Serum CEACAM1 readily followed tumor volumes and dropped dramatically two days after excision. In cases where the excision was complete, serum CEACAM1 gradually vanished from the circulation (Figure 2(a)), whereas in cases where tumor cells remained and tumor recurred, it reincreased in parallel to the elevation in tumor mass (Figures 2(b) and 2(c)). In the sham surgery group, tumors as well as serum CEACAM1 levels continued to increase gradually as expected (Figure 2(d)). We conclude that serum CEACAM1 sensitively and accurately reflects tumor burden in xenografted mice and may therefore serve as a novel biomarker for the monitoring of melanoma tumor burden and progression.

### 3.3. Baseline sCEACAM1 and LDH Levels Are Higher in Patients Who Fail to Respond to Treatment

**Figure 3:**
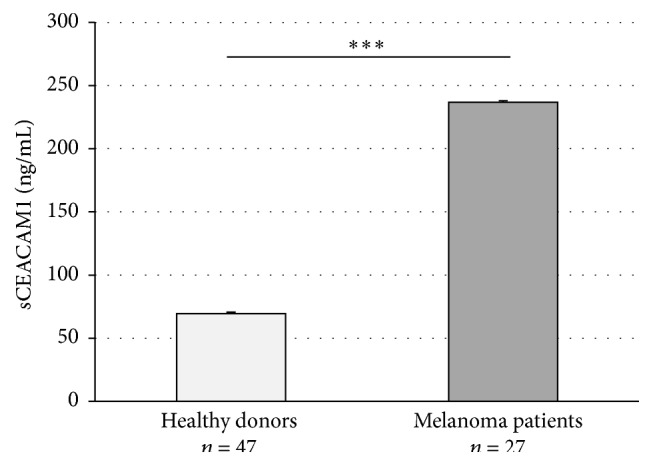
Serum CEACAM1 in healthy donors and melanoma patients. sCEACAM1 levels were assayed in the serum of 47 normal, healthy volunteers and in 27 melanoma patients. Statistics were assayed by regular Student's *t*-test.

**Figure 4:**
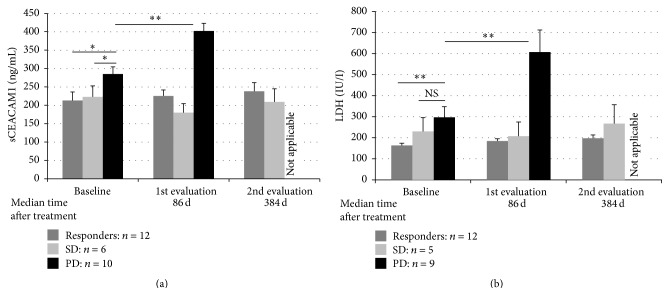
sCEACAM1 and LDH are elevated in melanoma patients who fail to respond to immunotherapy. sCEACAM1 (a) and LDH (b) levels were measured in clinically derived serum samples of AJCC stage IV metastatic melanoma patients that underwent immunotherapy with tumor infiltrating lymphocytes before as well as at 2 points after treatment (except for PD patients who passed away before the later time point). Statistics were assayed by regular Student's *t*-test.

In order to test the potential value of serum CEACAM1 for monitoring disease progression and response to treatment in melanoma patients, a retrospective longitudinal clinical trial was performed. We used a cohort of 27 AJCC stage IV melanoma patients that underwent immunotherapy with tumor infiltrating lymphocytes (TIL) [32, 33]. As expected [17, 27], serum CEACAM1 average levels were significantly (*P* < 0.001) higher in the whole cohort of melanoma patients as compared with healthy volunteers (Figure 3). In our clinical study, the response to treatment was evaluated starting from 30 days following TIL administration by radiological examination according to RECIST 1.0 criteria. Strikingly, there was a significant difference in the baseline (pretreatment) levels of serum CEACAM1 in between the patients (Figure 4(a), left columns). At this pretreatment time point, serum CEACAM1 was higher by 39% and 34% in patients that were later found to be not responding to treatment (PD) as opposed to responders and stable disease patients (285 versus 204 and 213 ng/mL, resp., *P* = 0.03). Furthermore, 26 out of the 27 patients were assayed for LDH at the same time point. Examining LDH values (Figure 4(b), left columns), we noticed that LDH was significantly elevated in PD patients as opposed to responders but not significantly in PD as opposed to SD patients. Though further examinations with a larger cohort are warranted, this result may point to the prognostic value of serum CEACAM1 in predicting response to adoptive transfer cell therapy or potentially other forms of immunotherapy.

### 3.4. sCEACAM1 and LDH Levels Are Elevated following Therapy in PD Patients Only

We continued by examining the changes in serum CEACAM1 and LDH following adoptive transfer cell therapy at 2 time points: 1.5–3.5 months (median: 86.5 days) and 11–18 months (median: 348 days) after TIL administration (Figures 4(a) and 4(b), middle and right columns). Unfortunately, PD patients passed away before the second posttreatment checkpoint could be achieved. We found that in patients who manifested disease progression (PD patients) serum CEACAM1 was significantly (*P* = 0.001) increased following treatment (a 40% increase, from 285 to 401 ng/mL in ~4 months). These results are in line with the xenograft evidences on the direct correlation between serum CEACAM1 and tumor burden. As expected, LDH values also increased following treatment (from 297 to 607 IU/I, *P* = 0.005). In the other groups of patients that did not show clinical deterioration, that is, the SD patients and the responders (CR and PR), there was no significant change in serum CEACAM1 (Figure 4(a)) or LDH (Figure 4(b)) over the ~17-month follow-up period.

### 3.5. The Changes in sCEACAM1 following Therapy Correlate with Those in LDH

**Figure 5:**
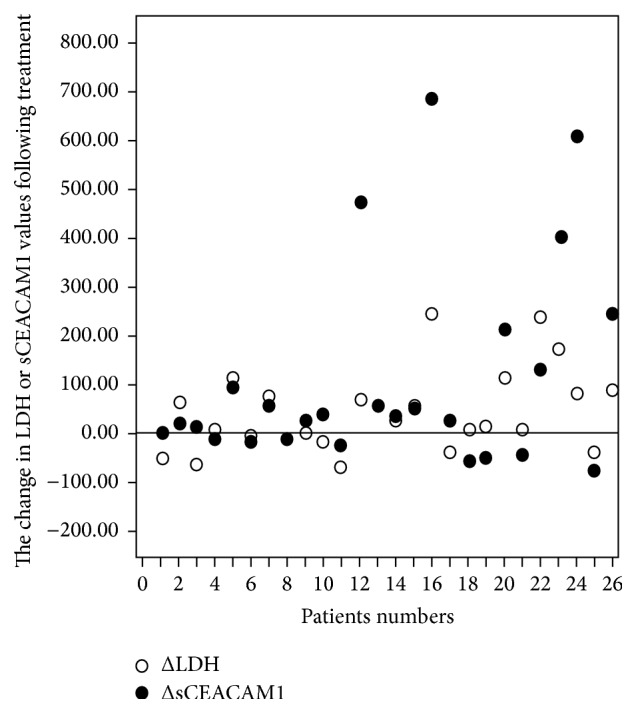
The changes in serum CEACAM1 in patients responding to treatment correlate with those in LDH. sCEACAM1 and LDH levels were tested at the same time points before and following immunotherapy for 26 AJCC stage IV patients. The baseline, pretreatment levels of sCEACAM1 (or LDH) were then subtracted from posttreatment levels and plotted as black (sCEACAM1) or white (LDH) circles for each of the patients.

We continued by assessing the relationships between serum CEACAM1 and LDH in response to TIL immunotherapy. We chose to relate to the 1st posttreatment values, which were available for all patients' subgroups, and assayed the changes in serum CEACAM1 (ΔsCEACAM1) and LDH (ΔLDH) at this time point as compared to baseline levels. Importantly, assessing the changes by the nonparametric Spearman correlation, we found a significant (Spearman coefficient = 0.764; significance 2-tailed = 0) correlation between ΔLDH and ΔsCEACAM1 (Figure 5 and Table 1).

Another parameter we tested is the time intervals from baseline to the 1st posttreatment time points, which ranged in between patients (baseline: 43–103 days before TIL treatment with median of 49.5 days and 1st posttreatment point at 48–102 days after treatment with median of 86.5 days). Specifically, we asked whether the differences in time intervals between patients (Δdays) could affect the obtained ΔsCEACAM1 and ΔLDH values. Using Spearmen correlation, we found that ΔLDH and ΔsCEACAM1 were not correlated with Δdays (i.e., were not increased as Δdays was increased; Table 1), supporting the strength of our findings.

Altogether, the results presented in this study imply a direct correlation between elevation in serum CEACAM1 levels, disease progression, and response to treatment and strengthen the prognostic value of sCEACAM1 in melanoma.

## 4. Discussion

Despite recent progression in the field of antimelanoma therapies, the prognosis of malignant melanoma patients still presents a clinical challenge as reliable biomarkers are scarce. LDH, which is mainly secreted from dead or damaged cells (concurring with tumor burden), is the strongest predictive serological marker for melanoma. It is incorporated in the TNM melanoma staging together with tumor thickness, mitotic rate, ulceration, and the presence of metastases [35–37]. Other reported candidate biomarkers include VEGF, tyrosinase, osteopontin, YKL-40, S100B, IL-8, and Cox-2. These, however, are also manifested by normal cells and under other malignancies (infectious disease, liver and renal injuries, autoimmunity, etc.) and therefore manifest undesired level of false-positive readouts [38–44]. Moreover, the existing markers are not suitable for deciphering specific subsets of patients and dictating therapeutic choices, such as patients who will benefit from novel immuno- and targeted therapies (and their combinations) or early-stage patients who are at high risk of relapse [1]. Therefore, urged by the generation of novel therapies, there is mandatory need for the discovery of melanoma biomarkers. Here we focused on a novel emerging melanoma biomarker, serum CEACAM1.

In this work we demonstrate that serum CEACAM1 sensitively reflects tumor volume in mice xenografted with human melanoma (Figures 1 and 2). This is in line with our previous results that serum CEACAM1 correlates with melanoma cell number in culture [17]. Importantly, serum CEACAM1 could be detected in mice even at minimal tumor volume of 14 mm^3^ which may imply on its prognostic value as an early diagnosis of melanoma. Indeed, it was shown that the changes in sCEACAM1 in non-small-cell lung cancer patients are more pronounced in early than in advanced tumors [31]. Moreover, in a large prospective study on pancreatic cancer patients, sCEACAM1 was found to be one of the earliest to be detected at significantly altered levels up to 35 months prior to diagnosis [28].

We have previously shown in two independent retrospective clinical studies that serum CEACAM1 is significantly higher in melanoma patients from different AJCC stages who show evidence of disease at the time of sampling, as compared to patients with no evident disease and healthy volunteers [17, 27] (Figure 3). Moreover, we found that serum CEACAM1 inversely correlates with survival [17, 27] and can stratify melanoma patients with evidence of disease into two prognostic groups with different survival rates [17]. In this study we continued using a different clinical scenario and explored AJCC stage IV melanoma patients that were subjected to adoptive cell transfer immunotherapy with tumor infiltrating lymphocytes (TIL ACT) after being refractory to all other treatments. We found that in patients who have not responded to treatment and continued to manifest progressive disease serum CEACAM1 was significantly (*P* = 0.001) higher as compared with responders and stable disease patients (Figure 4(a)). Collectively, these results show that serum CEACAM1 reflects tumor burden, disease progression, and survival.

Notably, serum CEACAM1 has not changed in responders following treatment in the two time checkpoints tested (Figure 4(a)). It may be that the expected decrease in sCEACAM1 in these patients was masked by CEACAM1 that was secreted from other cells so that the net serum CEACAM1 levels were balanced. Indeed, serum CEACAM1 is secreted from normal cells and is readily detected in the sera of healthy volunteers [17]. Moreover, it was demonstrated that apoptosis could induce cleavage of the intracellular and extracellular domains of CEACAM1, resulting in an increased level of serum CEACAM1 [45]. It may also be that at later time points, which were not checked, a change in serum CEACAM1 could be observed in responders.

Interestingly, when examining the baseline levels of serum CEACAM1, which were measured ~86 days before treatment, we found that serum CEACAM1 was significantly (*P* = 0.03) higher in PD patients, who fail to respond to treatment, as opposed to SD and responders (Figure 4(a), baseline). Noteworthy, in our experimental set-up, the same patients did not exhibit a statistically significant difference in LDH values in between PD and SD patients (Figure 4(b), baseline). We tested a battery of cytokines, including IL-8, TNF*α*, MIP1, MCP1, IL-4, and IL-17a, in pretreatment (basal) serum samples of melanoma patients who responded to TIL treatment (*n* = 8) as compared with patients who failed to respond (*n* = 8). Unfortunately, no significant differences were found between the groups in all cytokines tested (data not shown). These results point on possible prognostic value of serum CEACAM1 in predicting response to immunotherapy.

Collectively, our results show the prognostic value of serum CEACAM1 in monitoring tumor burden and disease progression. Though additional studies in larger cohorts and various therapeutic scenarios are warranted, they imply on the possible importance of serum CEACAM1 in early detection of melanoma and in prediction of response to immunotherapy.

## 5. Conclusions and Clinical Relevance

In this study we demonstrate that serum CEACAM1 (sCEACAM1) levels are correlated with tumor burden in immune-deficient mice xenografted with human melanoma. Moreover, in a clinical retrospective study (*n* = 28) we show that sCEACAM1 is increased only in patients who failed to respond to adoptive cell transfer therapy with tumor infiltrating lymphocytes and manifested progressive disease deterioration (PD). Strikingly, these patients were characterized by a higher pretreatment sCEACAM1, as compared with SD and responders. Moreover, the changes in post- versus pretreatment sCEACAM1 correlate with those in LDH. Altogether, these results imply on the prognostic value of sCEACAM1 in monitoring tumor burden, disease progression, and response to immunotherapy.

## Figures and Tables

**Table 1:** The correlations between ΔsCEACAM1, ΔLDH, and Δdays in melanoma patients.

	ΔLDH versus ΔsCEACAM1	ΔLDH versus Δdays	ΔsCEACAM1 versus Δdays
Correlation coefficient	0.764	0.19	0.202
Significance (2-tailed)	0	0.354	0.323

Spearman nonparametric correlation was used to assess the relationships between the changes of sCEACAM1 and LDH following treatment and between the differences in time intervals of testing points between patients and each of the markers.
